# Early Prediction of Sepsis-Related Coagulopathy: A Comparative Evaluation of Clinical Scoring Systems in the Emergency Department

**DOI:** 10.3390/jcm15114199

**Published:** 2026-05-29

**Authors:** Martina Petrucci, Stefania Gemma, Ivan Iozzia, Davide Antonio Della Polla, Nicola Bonadia, Gianluca Tullo, Piergiacomo Maria Cacciamani Fanelli, Antonio Gasbarrini, Francesco Franceschi, Marcello Covino

**Affiliations:** 1 Emergency Department, Fondazione Policlinico Universitario A. Gemelli IRCCS, 00168 Rome, Italy; martina.petrucci@policlinicogemelli.it (M.P.); davideantonio.dellapolla@policliicogemelli.it (D.A.D.P.); nicola.bonadia@policlinicogemelli.it (N.B.); gianluca.tullo@guest.policlinicogemelli.it (G.T.); francesco.franceschi@policlinicogemelli.it (F.F.); marcello.covino@policlinicogemelli.it (M.C.); 2 Faculty of Medicne, Università Cattolica Del Sacro Cuore, 00168 Rome, Italy; ivan.iozzia01@icatt.it (I.I.); pmg.cacciamani@gmail.com (P.M.C.F.); antonio.gasbarrini@policlinicogemelli.it (A.G.); 3 Department of International Medicine and Gastroenterolgy, Fondazione Policlinico Universitario A. Gemelli IRCCS, 00168 Rome, Italy

**Keywords:** sepsis-induced coagulopathy (SIC), emergency department, SIC score, JAAM-DIC score, ISTH overt DIC score, Endothelial Activation and Stress Index (EASIX)

## Abstract

**Background**: Early recognition of sepsis-related coagulation derangements may support timely risk stratification in emergency department (ED) patients with suspected infection. Evidence comparing coagulation-oriented scores in unselected “suspected sepsis” populations remains limited, particularly for predicting progression to sepsis, overt thrombotic/hemorrhagic events, and mortality. **Methods**: We conducted a single-center retrospective cohort study including consecutive ED patients evaluated for sepsis, suspected sepsis, or infection (January 2016–December 2024). Patients with overt disseminated intravascular coagulation (DIC) at admission were excluded. The Sepsis-Induced Coagulopathy (SIC), Japanese Association for Acute Medicine DIC (JAAM-DIC), International Society on Thrombosis and Haemostasis (ISTH) score, and Endothelial Activation and Stress Index (EASIX; and log2-EASIX) were calculated at admission. Outcomes were progression to confirmed sepsis (Sepsis-3), symptomatic coagulopathy (thrombotic and/or hemorrhagic events), and in-hospital mortality. Discrimination was assessed by ROC analysis (AUC with 95% CI) and clinical utility by decision curve analysis (DCA). **Results**: Among 11.274 patients without overt DIC, all scores showed moderate discrimination for mortality and sepsis progression. SIC performed best for in-hospital mortality (AUC 0.701, 95% CI 0.692–0.710) and for progression to confirmed sepsis (AUC 0.672, 95% CI 0.663–0.681). Prediction of overt thrombotic/hemorrhagic complications was poor across scores; ISTH yielded the highest (yet modest) AUCs for thrombotic events (0.614, 95% CI 0.604–0.623), hemorrhagic events (0.545, 95% CI 0.535–0.554), and combined coagulopathy (0.605, 95% CI 0.596–0.614). DCA suggested net benefit at low-to-intermediate threshold probabilities, particularly for SIC (and JAAM2) in early decision ranges. In the confirmed sepsis subgroup, discrimination attenuated overall; for combined coagulopathy, SIC showed the highest AUC (0.558, 95% CI 0.518–0.597). **Conclusions**: In ED patients with suspected sepsis and no overt DIC at presentation, SIC provides the strongest performance for early prognostic stratification (mortality and sepsis progression), whereas none of the evaluated scores reliably predict clinically overt thrombotic or hemorrhagic complications.

## 1. Introduction

Sepsis is a time-critical syndrome and a major driver of preventable in-hospital morbidity and mortality. In everyday acute care, clinicians frequently evaluate patients with suspected infection, yet only a subset will progress to confirmed sepsis or develop organ dysfunction and other severe complications.

In patients presenting with suspected infection, early identification of those at risk of clinical deterioration is crucial, as delayed or inappropriate treatment is associated with worse outcomes. However, distinguishing patients who will progress to confirmed sepsis or develop severe complications remains challenging in the early stages, when clinical signs may be nonspecific [1,2,3,4].

Coagulation abnormalities represent a central component of sepsis pathophysiology. Sepsis-induced coagulopathy (SIC) reflects early dysregulation of the interaction between inflammation, endothelial activation, and thrombin generation, contributing to microvascular dysfunction and organ failure. These alterations may precede overt clinical deterioration and therefore offer a potential opportunity for early risk stratification [5,6,7,8,9].

Several scoring systems have been developed to quantify sepsis-related coagulation disturbances. The International Society on Thrombosis and Haemostasis (ISTH) overt DIC score was designed to diagnose established disseminated intravascular coagulation [10,11,12], whereas the Sepsis-Induced Coagulopathy (SIC) score was proposed to identify earlier, sepsis-specific coagulation abnormalities [13,14,15]. The Japanese Association for Acute Medicine (JAAM-DIC) score has been validated mainly in septic populations, particularly for mortality prediction [16,17,18]. More recently, the Endothelial Activation and Stress Index (EASIX) has emerged as a surrogate marker of endothelial dysfunction and has shown prognostic relevance in critically ill patients [19,20,21].

Most validation studies have focused on selected cohorts with confirmed sepsis, primarily evaluating mortality or organ dysfunction. Data are limited regarding the performance of these coagulation-related scores in unselected patients presenting with suspected sepsis, and their ability to predict progression to sepsis, clinically overt coagulopathy (thrombotic or hemorrhagic events), or in-hospital mortality remains uncertain [22,23,24,25].

From an antimicrobial stewardship perspective, early risk stratification may help balance timely antibiotic initiation with avoidance of unnecessary escalation and resource overuse [26]. In this retrospective study, we evaluated consecutive patients presenting with sepsis or suspected sepsis over nine years, excluding those with overt DIC at presentation. We assessed the baseline performance of SIC, JAAM-DIC, ISTH, and EASIX scores in predicting progression to confirmed sepsis, development of symptomatic coagulopathy, and in-hospital mortality using receiver operating characteristic analysis and decision curve analysis.

By clarifying the prognostic and clinical utility of these scores in a real-world population with suspected infection, our study aims to inform early risk-adapted management strategies in acute infectious settings.

## 2. Methods and Statistical Analysis

### 2.1. Study Design and Population

This is a monocentric retrospective cohort study of consecutive patients admitted to the Emergency Department between January 2016 and December 2024 with sepsis, suspected sepsis, or clinically suspected infection at initial evaluation. Patients were identified based on emergency department documentation, clinical suspicion of infection prompting diagnostic work-up and/or initiation of antimicrobial therapy, according to routine clinical practice.

A total of 11,672 patients were screened. The study was intentionally designed to evaluate the prognostic and clinical utility of coagulation-related scores in a real-world population undergoing early sepsis assessment, where definitive confirmation of infection is frequently unavailable at presentation and therapeutic decisions must often be made under conditions of diagnostic uncertainty.

Patients with overt disseminated intravascular coagulation (DIC) at admission were excluded. Of the 11,672 screened patients, 398 were excluded because of overt DIC at presentation, yielding a final study population of 11,274 patients without overt DIC for the primary analyses.

To address potential heterogeneity related to the inclusion of patients without subsequently confirmed infection, predefined subgroup analyses were additionally performed in patients with confirmed sepsis.

### 2.2. Data Collection

Demographic, clinical, and laboratory data were collected retrospectively from electronic medical records. Baseline variables were recorded at admission and included age, comorbidity burden (Charlson Comorbidity Index), major comorbidities, vital signs, oxygenation parameters (PaO_2_/FiO_2_), and coagulation-related laboratory tests (platelet count, INR, aPTT, fibrinogen, and D-dimer).

At baseline, the following scores were calculated for each patient using routinely available clinical/laboratory parameters:

- Sepsis-Induced Coagulopathy (SIC) score
- Japanese Association for Acute Medicine DIC (JAAM-DIC) score
- International Society on Thrombosis and Haemostasis (ISTH) score
- Endothelial Activation and Stress Index (EASIX)
- log2-transformed EASIX (log2-EASIX)

### 2.3. Outcomes

The main outcomes of interest were:

- Progression to confirmed sepsis during hospitalization (among patients without confirmed sepsis at admission), according to Sepsis-3
- In-hospital mortality.
- Symptomatic coagulopathy, evaluated as:
  Thrombotic events;
  Hemorrhagic events;
  Combined coagulopathy endpoint (thrombotic and/or hemorrhagic events).

### 2.4. Statistical Analysis

Continuous variables are reported as median and interquartile range (IQR) and compared using univariate analysis by the Mann–Whitney U test.

Categorical variables are expressed as numbers and percentages and were compared using Chi-square test (with Fisher’s test if appropriate).

The discriminative performance of SIC, JAAM-DIC, ISTH, EASIX, and log2-EASIX for each clinical outcome was evaluated using receiver operating characteristic (ROC) curve analysis. Area under the ROC curve (AUC) values were calculated with 95% confidence intervals (CIs). Pairwise comparisons between ROC curves were performed using the DeLong method.

To assess potential clinical usefulness beyond discrimination alone, decision curve analysis (DCA) was performed for progression to sepsis, combined coagulopathy, and in-hospital mortality in the non-overt DIC population, and for mortality and combined coagulopathy in the confirmed sepsis subgroup. Net benefit was examined across clinically relevant threshold probabilities and compared with the default strategies of “treat-all” and “treat-none.”

Multivariable logistic regression analyses were performed to assess the independent association between the best-performing coagulation score (based on ROC analysis and DCA) and each clinical outcome, adjusting for relevant clinical and demographic covariates.

All tests were two-sided, and *p* values < 0.05 were considered statistically significant. Statistical analyses were performed using IBM SPSS statistics for Windows, Version 25 (IBM Corp., Armonk, NY, USA) and MedCalc Statistical Software version 19.2.1 (MedCalc, Ostend, Belgium).

## 3. Results

Baseline values of all evaluated coagulation scores (SIC, JAAM, ISTH, EASIX, and log2-EASIX), measured at admission, were analyzed in relation to clinical outcomes. Baseline characteristics of the non-overt DIC study population are shown in Table 1.

The results of the analysis of the characteristics of the subgroups included in the study can be found in Appendix A.

In univariate analyses, higher SIC and EASIX scores were significantly associated with mortality, while their association with clinically overt thrombotic or hemorrhagic events was weak or absent. ISTH and JAAM scores showed limited or inconsistent associations across outcomes, particularly for bleeding and thrombotic manifestations.

Patients who subsequently progressed to sepsis showed significantly higher baseline coagulation scores compared with those who did not, despite the absence of overt DIC at presentation.

### 3.1. Performance of Coagulation Scores in a Non-Selected Population of Suspected Septic Patients

#### 3.1.1. ROC Analysis for Mortality

**Figure 1:**
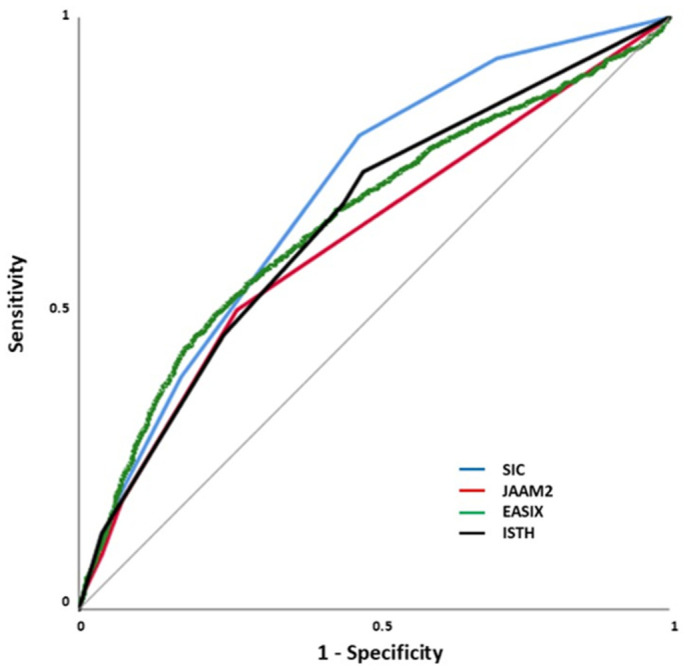
ROC for all causes of in-hospital mortality. Receiver operating characteristic (ROC) curves of SIC, JAAM-DIC, ISTH, and EASIX scores for predicting in-hospital mortality. AUCs (95% CI): SIC 0.701 (0.692–0.710), ISTH 0.655 (0.646–0.664), EASIX 0.657 (0.648–0.666), log2-EASIX 0.657 (0.648–0.666), JAAM-DIC 0.625 (0.616–0.634). Abbreviations: SIC, Sepsis-Induced Coagulopathy; JAAM2, Japanese Association for Acute Medicine2; ISTH, International Society on Thrombosis and Haemostasis; EASIX, Endothelial Activation and Stress Index.

All coagulation scores demonstrated a moderate ability to predict mortality (Figure 1).

The SIC score showed the highest discriminative performance, with an AUC of 0.701 (95% CI 0.692–0.710), significantly outperforming ISTH, JAAM, EASIX, and log2-EASIX scores (all pairwise comparisons *p* < 0.0001).

EASIX, log2-EASIX, and ISTH scores showed similar performance (AUC ~ 0.65), while JAAM had the lowest discriminative ability (AUC 0.625).

#### 3.1.2. ROC Analysis for Progression to Sepsis

**Figure 2:**
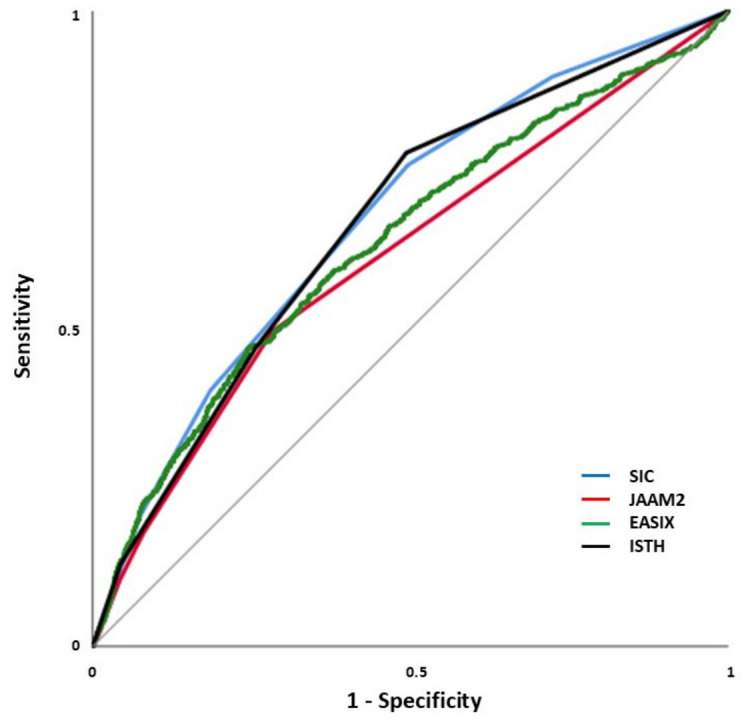
ROC for progression to sepsis. Receiver operating characteristic (ROC) curves of SIC, JAAM-DIC, ISTH, and EASIX scores for predicting progression to confirmed sepsis in ED patients with suspected infection. AUCs (95% CI): SIC 0.672 (0.663–0.681), ISTH 0.664 (0.655–0.673), EASIX 0.634 (0.625–0.643), log2-EASIX 0.634 (0.625–0.643), JAAM-DIC 0.612 (0.603–0.622). Abbreviations: SIC, Sepsis-Induced Coagulopathy; JAAM2, Japanese Association for Acute Medicine2; ISTH, International Society on Thrombosis and Haemostasis; EASIX, Endothelial Activation and Stress Index.

All coagulation scores showed a moderate ability to predict progression to sepsis (Figure 2).

The SIC score showed the highest AUC (0.672; 95% CI 0.663–0.681), followed closely by the ISTH score (0.664; 95% CI 0.655–0.673), with no significant difference between the two.

Both scores significantly outperformed JAAM, while EASIX and log2-EASIX showed intermediate performance.

#### 3.1.3. ROC Analysis for Thrombotic Events

The ability of coagulation scores to predict thrombotic complications was limited.

The ISTH score showed the highest AUC (0.614; 95% CI 0.604–0.623), although its discriminative ability remained modest.

EASIX and JAAM scores showed poor performance (AUCs < 0.56), while the SIC score demonstrated no discriminative ability, with an AUC close to random classification (0.509; 95% CI 0.500–0.519).

Pairwise comparisons confirmed that ISTH significantly outperformed all other scores (*p* < 0.0001), whereas SIC performed significantly worse than all comparators.

#### 3.1.4. ROC Analysis for Hemorrhagic Events

All evaluated scores showed poor discrimination for hemorrhagic complications.

The ISTH score again showed the highest AUC (0.545; 95% CI 0.535–0.554), though its performance was clinically limited.

JAAM and SIC scores showed similarly low performance (AUC ~ 0.53), while EASIX and log2-EASIX scores demonstrated no discriminative ability (AUC ~ 0.50).

Although some pairwise differences reached statistical significance, none of the evaluated scores demonstrated clinically meaningful predictive accuracy for bleeding events.

#### 3.1.5. ROC Analysis for Combined Coagulopathy Endpoint

**Figure 3:**
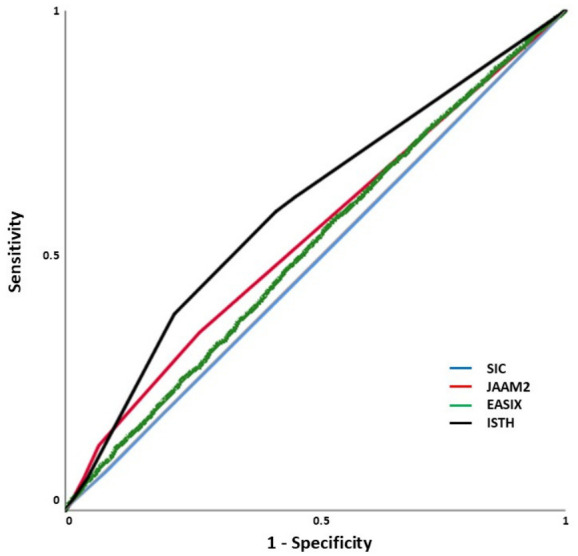
ROC for symptomatic coagulopathy. Receiver operating characteristic (ROC) curves of SIC, JAAM-DIC, ISTH, and EASIX scores for predicting symptomatic coagulopathy (thrombotic and/or hemorrhagic events). AUCs (95% CI): ISTH 0.605 (0.596–0.614), JAAM-DIC 0.549 (0.540–0.559), EASIX 0.527 (0.517–0.536), log2-EASIX 0.527 (0.517–0.536), SIC 0.503 (0.494–0.513). Abbreviations: SIC, Sepsis-Induced Coagulopathy; JAAM2, Japanese Association for Acute Medicine2; ISTH, International Society on Thrombosis and Haemostasis; EASIX, Endothelial Activation and Stress Index.

When thrombotic and hemorrhagic events were analyzed as a combined endpoint, all scores showed limited predictive performance (Figure 3).

The ISTH score demonstrated the highest AUC (0.605; 95% CI 0.596–0.614), whereas JAAM and EASIX scores showed poor discrimination.

The SIC score again showed no discriminative ability, with an AUC close to 0.50.

#### 3.1.6. Summary of ROC Performance Across Outcomes

Across all analyses, a consistent pattern emerged:

- Coagulation scores, particularly SIC, demonstrated the strongest performance for systemic and prognostic outcomes (mortality and sepsis progression);
- All scores showed poor predictive accuracy for clinically overt thrombotic or hemorrhagic events, even when analyzed as a combined endpoint.

In Table 2, we provide a comparison of the performance of the evaluated scores across clinical outcomes.

#### 3.1.7. Decision Curve Analysis for Sepsis Evolution

**Figure 4:**
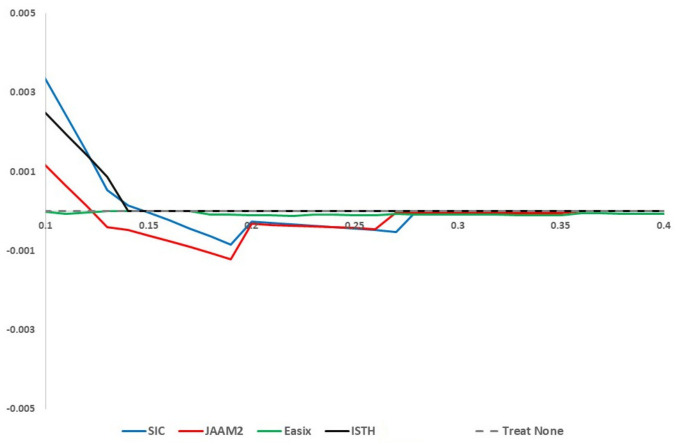
Decision curve analysis (DCA) of SIC, JAAM-DIC, ISTH, and EASIX scores for predicting progression to confirmed sepsis, showing net benefit across a range of threshold probabilities compared with “treat-all” and “treat-none” strategies. Abbreviations: SIC, Sepsis-Induced Coagulopathy; JAAM2, Japanese Association for Acute Medicine2; ISTH, International Society on Thrombosis and Haemostasis; EASIX, Endothelial Activation and Stress Index.

The decision curve analysis for progression to confirmed sepsis demonstrates that all four scoring systems: SIC, JAAM2, EASIX, and ISTH, provide positive net benefit across low-threshold probabilities compared with both default strategies (“treat-all” and “treat-none”). The clinical utility of these models is most evident in the low-risk range, approximately 1–10%, thresholds at which ED clinicians would reasonably consider empirical antimicrobial treatment or closer monitoring (Figure 4).

Among the evaluated scores, SIC and JAAM2 show the most consistent and sustained net benefit within this low-threshold region. Their curves remain above both default strategies across the range in which early antimicrobial initiation, hemodynamic optimization, or closer monitoring would reasonably be considered. EASIX demonstrates positive net benefit at very low thresholds but shows earlier attenuation compared with SIC and JAAM2, suggesting more limited incremental utility as predicted probability increases. ISTH displays an intermediate profile, with positive net benefit at low thresholds but a tendency to plateau earlier at mid-range probabilities.

Importantly, the treat-all strategy becomes inferior beyond very low thresholds, indicating that indiscriminate activation of sepsis-directed management would lead to unnecessary interventions. In contrast, the score-based approaches maintain positive net benefit within clinically actionable ranges, supporting their potential role in structured risk stratification during early sepsis evaluation.

#### 3.1.8. Decision Curve Analysis for Combined Coagulopathy Endpoint

For the composite outcome of symptomatic coagulopathy (bleeding or thrombosis), all four scoring systems outperform the treat-none strategy across a broad range of threshold probabilities. The greatest clinical utility is observed in the low-to-intermediate probability range, approximately 1–25%, corresponding to clinically actionable decisions such as intensified coagulation monitoring, hematologic consultation, or individualized.

SIC and JAAM2 exhibit highly overlapping and consistently favorable net benefit curves throughout this interval, suggesting robust performance in identifying patients at increased risk of clinically relevant coagulopathy. EASIX also provides positive net benefit, although with earlier attenuation and greater variability compared with SIC and JAAM2. The ISTH score demonstrates a more limited incremental advantage, with its curve approaching that of the treat-none strategy at intermediate thresholds.

As threshold probabilities increase beyond approximately 25–30%, the net benefit of all models declines, reflecting the rarity of very high predicted risks and the increasing penalty associated with false-positive classifications. Notably, the treat-all strategy becomes progressively harmful at moderate thresholds, underscoring the importance of selective rather than indiscriminate intervention.

**Figure 5:**
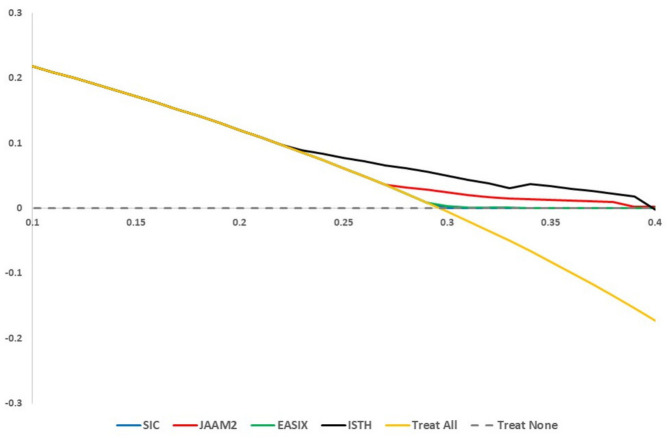
Decision curve analysis (DCA) of SIC, JAAM-DIC, ISTH, and EASIX scores for predicting symptomatic coagulopathy (thrombotic and/or hemorrhagic events), illustrating net clinical benefit across threshold probabilities. Abbreviations: SIC, Sepsis-Induced Coagulopathy; JAAM2, Japanese Association for Acute Medicine 2; ISTH, International Society on Thrombosis and Haemostasis; EASIX, Endothelial Activation and Stress Index.

**Figure 6:**
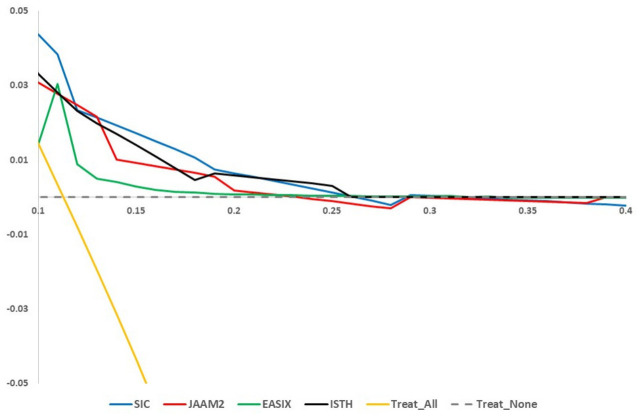
Decision curve analysis (DCA) of SIC, JAAM-DIC, ISTH, and EASIX scores for predicting in-hospital mortality, comparing net benefit with Treat-all and Treat-None strategies. Abbreviations: SIC, Sepsis-Induced Coagulopathy; JAAM2, Japanese Association for Acute Medicine 2; ISTH, International Society on Thrombosis and Haemostasis; EASIX, Endothelial Activation and Stress Index.

**Figure 7:**
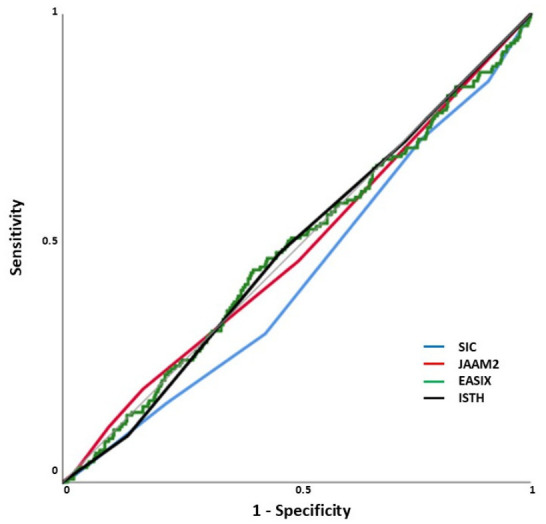
ROC analysis in septic patients for the combined coagulopathy endpoint. AUCs (95% CI): SIC 0.558 (0.518–0.597), JAAM-DIC 0.506, EASIX 0.505, ISTH 0.502. Abbreviations: SIC, Sepsis-Induced Coagulopathy; JAAM2, Japanese Association for Acute Medicine 2; ISTH, International Society on Thrombosis and Haemostasis; EASIX, Endothelial Activation and Stress Index.

Overall, these findings indicate that coagulation-based scores, particularly SIC and JAAM2, may offer clinically meaningful support in early identification of patients at risk of developing overt coagulopathy (Figure 5, Figure 6 and Figure 7).

#### 3.1.9. Decision Curve Analysis for In-Hospital Mortality

The decision curve analysis for in-hospital mortality reveals a more heterogeneous pattern, consistent with the multifactorial nature of death in septic patients. Across low-threshold probabilities, approximately 1–15%, SIC, JAAM2, and EASIX demonstrate positive net benefit compared with both default strategies, indicating potential utility for early prognostic assessment, escalation of care, ICU evaluation, or intensified monitoring.

Among the models, SIC maintains the most consistent net benefit across low and mid-range thresholds, while JAAM2 shows early positive utility followed by more rapid attenuation as thresholds increase. EASIX provides modest net benefit at low thresholds but does not maintain sustained superiority at higher decision probabilities. The ISTH score offers comparatively limited incremental value, with its curve approaching the treat-none strategy at lower thresholds than the other models.

As observed for the other outcomes, the treat-all strategy rapidly becomes harmful beyond low-probability cut-offs, emphasizing that universal escalation of care based solely on baseline suspicion would result in overtreatment. Conversely, model-guided stratification, particularly using SIC, appears to provide incremental clinical utility in the range where early mortality risk estimation is most actionable.

Collectively, these results support the role of sepsis-related coagulation scores as adjunctive tools for early risk stratification, while also highlighting their limited usefulness at higher probability thresholds and in late-stage decision contexts.

### 3.2. Performance of Coagulation Scores in Septic Patients

In the subgroup of patients with confirmed sepsis, ROC analyses were performed to assess the ability of sepsis-related coagulation scores to predict in-hospital mortality and the subsequent development of coagulopathy.

#### 3.2.1. ROC Analysis for Combined Coagulopathy Endpoint

For the composite coagulopathy outcome, the SIC score demonstrated the highest discriminative performance (AUC 0.558, 95% CI 0.518–0.597), whereas JAAM-DIC, EASIX, and ISTH scores showed limited discrimination, with AUCs close to 0.50. Pairwise comparisons confirmed that SIC performed significantly better than JAAM-DIC and EASIX, while the difference versus the ISTH score showed a non-significant trend (Figure 3).

#### 3.2.2. ROC Analysis for Mortality

**Figure 8:**
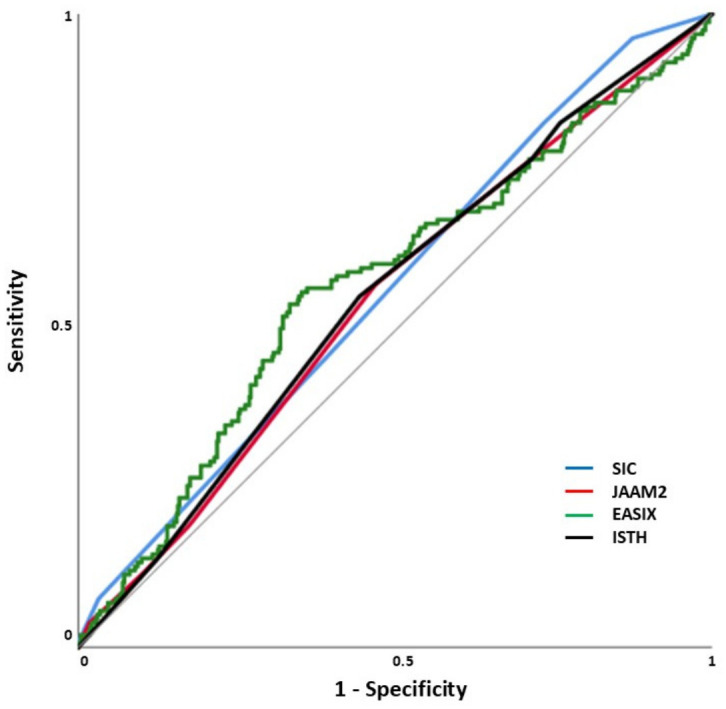
ROC analysis in septic patients for in-hospital mortality. AUCs: EASIX 0.574, SIC 0.566, ISTH 0.554, JAAM-DIC 0.548. Abbreviations: SIC, Sepsis-Induced Coagulopathy; JAAM2, Japanese Association for Acute Medicine 2; ISTH, International Society on Thrombosis and Haemostasis; EASIX, Endothelial Activation and Stress Index.

In contrast, for in-hospital mortality, all evaluated scores showed modest and overlapping discriminative ability, with no statistically significant differences observed among SIC, JAAM-DIC, EASIX, and ISTH scores (Figure 8). These findings indicate an attenuation of prognostic discrimination when analyses are restricted to patients with established sepsis.

#### 3.2.3. Decision Curve Analysis for Coagulopathy

**Figure 9:**
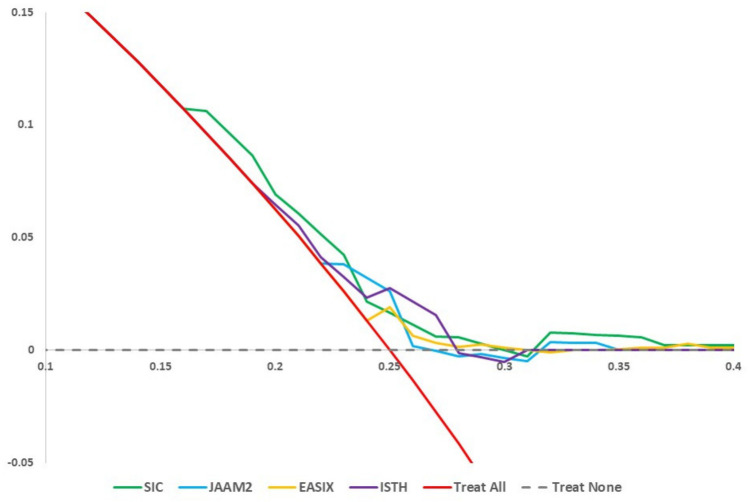
Decision Curve Analysis in septic patients for coagulopathy. Abbreviations: SIC, Sepsis-Induced Coagulopathy; JAAM2, Japanese Association for Acute Medicine 2; ISTH, International Society on Thrombosis and Haemostasis; EASIX, Endothelial Activation and Stress Index.

In the decision curve analysis assessing the ability of four sepsis-related coagulation scores to predict the subsequent development of coagulopathy, the SIC, JAAM-DIC (JAAM2), EASIX, and ISTH scores all demonstrated positive net clinical benefit across low-to-moderate threshold probabilities compared with a “treat-none” strategy (Figure 9).

Among them, the SIC and JAAM2 scores showed the most consistently favorable performance, providing the greatest net benefit within a threshold probability range of approximately 0.01–0.20. This clinically relevant interval corresponds to early or empiric management decisions, such as intensified monitoring, anticoagulation consideration, or hematologic consultation. Within this range, both scores also outperformed the “treat-all” strategy, indicating improved identification of patients at higher risk while limiting unnecessary interventions.

The EASIX score demonstrated modest net benefit over a narrower range of thresholds, whereas the ISTH score provided limited incremental value and, at several thresholds, performed similarly to a “treat-none” strategy. At higher threshold probabilities (>0.20), net benefit declined across all models, reflecting the low prevalence of high-probability events and the increasing impact of false-positive classifications.

#### 3.2.4. Decision Curve Analysis for In-Hospital Mortality

**Figure 10:**
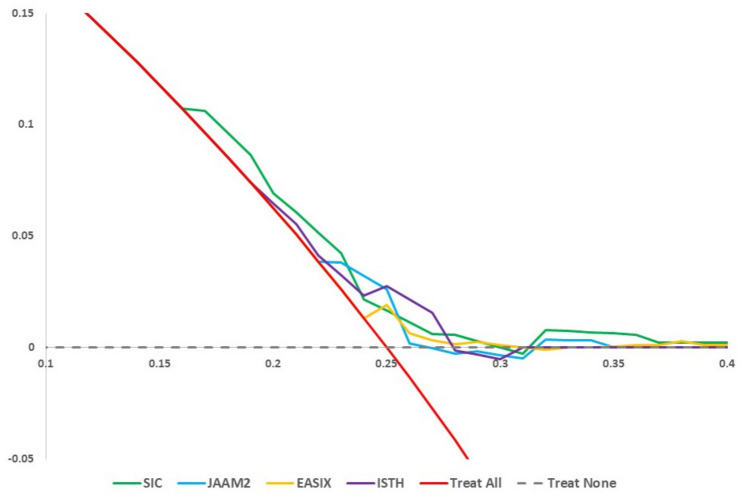
Decision Curve Analysis in septic patients for in-hospital mortality. Abbreviations: SIC, Sepsis-Induced Coagulopathy; JAAM2, Japanese Association for Acute Medicine 2; ISTH, International Society on Thrombosis and Haemostasis; EASIX, Endothelial Activation and Stress Index.

For in-hospital mortality, decision curve analysis revealed that the SIC, JAAM2, and EASIX scores provided positive net benefit across low-threshold probabilities (0.01–0.15), outperforming both “treat-all” and “treat-none” strategies (Figure 10). This suggests potential utility in early prognostic stratification and resource allocation, including escalation of care or closer clinical monitoring.

At intermediate thresholds (0.15–0.25), the SIC score maintained the highest net benefit, while JAAM2 and EASIX showed modest attenuation but remained superior to a “treat-none” approach. In contrast, the ISTH score consistently showed limited incremental benefit across most thresholds. At higher thresholds (>0.25), net benefit decreased for all models, consistent with the increasing penalty of false-positive decisions at more stringent cut-offs.

#### 3.2.5. Multivariate Analysis

To assess whether the observed associations persisted after adjustment for potential confounders, multivariable logistic regression analyses were performed. After adjustment for age, sex, Charlson Comorbidity Index, and baseline SOFA score, the SIC score was independently associated with an increased risk of progression to sepsis (OR 1.41, 95% CI 1.31–1.50; *p* < 0.001).

Similarly, after adjustment for age, sex, Charlson Comorbidity Index, and baseline SOFA score, higher SIC scores were independently associated with an increased risk of in-hospital mortality (OR 1.20, 95% CI 1.13–1.28; *p* < 0.001).

Finally, considering only the patients who progressed to overt sepsis, the SIC score at admission maintained an independent association with in-hospital mortality (OR 1.45, 95% CI 1.37–1.52; *p* < 0.001).

## 4. Discussion

In this large, unselected cohort of patients with suspected sepsis and without overt disseminated intravascular coagulation at presentation, we evaluated the performance of several validated sepsis-related coagulopathy scores across a broad spectrum of clinically relevant outcomes. Our results demonstrate that coagulation scores, particularly the Sepsis-Induced Coagulopathy (SIC) score, are effective tools for global risk stratification and prognostic assessment but show limited or no ability to predict clinically overt thrombotic or hemorrhagic complications.

The strongest and most consistent finding of this study is the robust performance of the SIC score for predicting mortality and progression to sepsis, even in patients without overt DIC at baseline. These findings support the concept that sepsis-related coagulopathy represents an early, systemic host response reflecting endothelial dysfunction, inflammation-driven coagulation activation, and microvascular impairment, rather than a direct precursor of clinically apparent hemostatic events [6,27].

The SIC score integrates platelet count, coagulation abnormalities, and organ dysfunction, thereby capturing both hemostatic activation and systemic illness severity [28]. This likely explains its superior prognostic performance compared with JAAM and ISTH scores, which were originally developed to identify overt or advanced DIC rather than early dysregulation of coagulation in sepsis. Because SIC incorporates components of the SOFA score, its prognostic value probably reflects not only coagulation abnormalities but also the overall severity of the septic host response, including endothelial dysfunction, inflammation, and early organ failure. Indeed, evidence from the literature shows that the JAAM-DIC score, proposed by the Japanese Association for Acute Medicine, appears to be more sensitive for the early detection of DIC in sepsis, comparable to the SIC score, whereas the ISTH-DIC score is more specific for advanced stages of the disease and shows a stronger correlation with clinical outcomes [16,22,23].

In contrast to its prognostic performance, the SIC score showed no discriminative ability for thrombotic, hemorrhagic, or combined coagulopathy endpoints, supporting the concept that SIC is primarily a severity and prognostic index rather than a specific predictor of overt hemostatic complications.

In the unselected population of patients with suspected sepsis, decision curve analysis demonstrated that all four scores (SIC, JAAM2, EASIX, and ISTH) provided positive net clinical benefit compared with the “treat-all” and “treat-none” strategies, particularly within low-threshold probabilities (approximately 1–15%), corresponding to the most clinically actionable decision range. For progression to confirmed sepsis, SIC and JAAM2 showed the most consistent and sustained net benefit at low thresholds, supporting their role in early diagnostic stratification. For symptomatic coagulopathy, net benefit extended across a broader range of intermediate thresholds (up to approximately 20–25%), with SIC and JAAM2 again demonstrating the most stable performance, while EASIX and ISTH exhibited earlier attenuation. In contrast, for in-hospital mortality, the curves were more heterogeneous, with SIC maintaining the most consistent net benefit and the other scores showing more modest and less sustained utility as thresholds increased. Overall, despite only moderate discrimination in ROC analyses, these findings indicate that the scores retain meaningful clinical utility for early risk stratification, particularly at low, clinically relevant decision thresholds.

These findings, consistent with recent evidence from the literature, highlight an important conceptual distinction: clinically overt thrombotic and hemorrhagic events are multifactorial phenomena. Their occurrence depends not only on systemic coagulation activation but also on local vascular factors, invasive procedures, immobilization, anticoagulant or antiplatelet therapy, transfusion practices, and individual bleeding risk. Indeed, recent evidence shows that SIC scores are associated with mortality but perform poorly in predicting thrombotic and hemorrhagic events, suggesting that they reflect overall severity of illness rather than specific vascular complications [29]. Moreover, the complex interplay between inflammation, endothelial dysfunction, and coagulation in sepsis further complicates the relationship between systemic coagulopathy and overt clinical events [6]. Finally, clinical cohorts have identified multiple independent risk factors for bleeding and thrombosis beyond coagulation activation alone, emphasizing the multifactorial nature of these outcomes in critically ill patients [30].

Our results are consistent with prior observations showing weak correlations between laboratory-defined coagulopathy and clinically evident thrombosis or bleeding in critically ill patients and underscore the limitations of using sepsis-related coagulation scores as surrogate markers of bleeding or thrombotic risk [31,32,33].

Previous studies validating SIC, JAAM, and ISTH scores primarily focused on mortality and organ dysfunction in selected septic populations. In these settings, SIC has consistently demonstrated superior prognostic performance compared with traditional DIC scores [22,24]. Our findings extend these observations by demonstrating that the prognostic value of SIC persists beyond the classic septic population, remaining valid in an unselected cohort of patients with suspected sepsis.

Data on the ability of coagulation scores to predict clinical thrombotic or hemorrhagic events are limited and inconsistent in the literature. Our study addresses this gap by systematically evaluating these outcomes and showing that none of the evaluated scores can reliably identify patients at risk for overt coagulopathy-related complications.

EASIX, originally developed as a marker of endothelial injury and microangiopathy [19,20], showed intermediate performance for mortality and sepsis progression but failed to predict bleeding or thrombotic events. This further supports the interpretation that endothelial injury and microvascular dysfunction are central to sepsis severity but are not direct determinants of macroscopic hemostatic complications.

When analyses were restricted to patients with established sepsis, discriminative performance for mortality was markedly attenuated, and differences among scores largely disappeared. This apparent loss of prognostic stratification likely reflects spectrum restriction, as well as the increasing influence of non-coagulation-related determinants of outcome in advanced disease stages.

Importantly, despite modest ROC performance in septic patients, decision curve analyses demonstrated that several scores, most notably SIC and JAAM-DIC, provided meaningful net clinical benefit across low-threshold probabilities. This finding indicates that even limited discrimination may translate into clinical usefulness when scores are applied to support early or empiric decisions, such as intensified monitoring, anticoagulation consideration, or timely specialist consultation.

Our results further reinforce a conceptual distinction between systemic coagulation dysregulation, as captured by laboratory-based scores, and clinically overt thrombotic or hemorrhagic events, which are multifactorial phenomena influenced by local vascular factors, invasive procedures, concomitant therapies, and individual bleeding or thrombotic risk. Consequently, sepsis-related coagulation scores appear better suited for early risk stratification and clinical guidance at low decision thresholds rather than for precise prediction of specific hemostatic events in patients with established sepsis.

From a clinical perspective, the early applicability of the SIC score in the Emergency Department may support timely risk stratification and early sepsis-oriented management, despite its only moderate predictive performance. Future integration of coagulation-based scores with machine learning approaches may further improve individualized prognostic assessment in sepsis.

## 5. Strength and Limitations

The main strengths of this study include the large sample size, the inclusion of an unselected population with suspected sepsis, and the use of clinically meaningful outcomes rather than laboratory-defined endpoints. However, several limitations must be acknowledged. The observational design precludes causal inference, and management strategies such as anticoagulation or transfusion were not standardized and may have influenced clinical outcomes. In addition, only baseline coagulation parameters were analyzed, and dynamic changes over time were not evaluated.

## 6. Conclusions

In conclusion, sepsis-related coagulation scores, particularly the SIC score, are effective markers of disease severity and prognosis in patients with suspected sepsis, even in the absence of overt DIC. However, these scores fail to predict clinically apparent thrombotic or hemorrhagic complications, reinforcing the concept that sepsis-related coagulopathy is primarily a manifestation of systemic dysfunction rather than a direct predictor of hemostatic events.

## Figures and Tables

**Table 1:** Baseline characteristics of the non-overt DIC study population.

Variable	Overall Population (*n* = 11,274)
Age, years	71 (58–81)
Charlson Comorbidity Index	4 (2–6)
Heart Failure, *n* (%)	1426 (12.6)
COPD, *n* (%)	1506 (13.4)
Diabetes, *n* (%)	1632 (14.5)
Dementia, *n* (%)	682 (6)
Cirrhosis, *n* (%)	441 (3.9)
Chronic kidney disease, *n* (%)	1078 (9.6)
Malignancy, *n* (%)	1695 (15)
Systolic blood pressure, mmHg	133 (116–150)
Mean arterial pressure, mmHg	96.7 (86.3–108.7)
Heart rate, bpm	88 (77–103)
PaO_2_/FiO_2_ ratio	411 (313–476)
Platelet count, ×10^9^/L	230 (175–302)
INR	1.07 (1.01–1.15)
aPTT, seconds	30.9 (27.2–35.4)
Fibrinogen, mg/dL	430 (333–585)
D-dimer, ng/mL	1251 (561–3346)
SIC score	2 (0–2)
JAAM-DIC score	0 (0–1)
ISTH score	1 (0–3)
EASIX	1.20 (0.71–2.28)
log2-EASIX	0.26 (−0.50–1.19)

Data are expressed as median [IQR] or *n* (%). Abbreviations: COPD, chronic obstructive pulmonary disease; PaO_2_/FiO_2_, arterial oxygen partial pressure to fractional inspired oxygen ratio; INR, international normalized ratio; aPTT, activated partial thromboplastin time; SIC, Sepsis-Induced Coagulopathy; JAAM-DIC, Japanese Association for Acute Medicine disseminated intravascular coagulation; ISTH, International Society on Thrombosis and Haemostasis; EASIX, Endothelial Activation and Stress Index.

**Table 2:** Discriminative performance of coagulation scores for clinical outcomes.

Outcome	Score	AUC	95% CI
**Mortality**	**SIC**	**0.701**	0.692–0.710
	ISTH	0.655	0.646–0.664
	EASIX	0.657	0.648–0.666
	log2-EASIX	0.657	0.648–0.666
	JAAM	0.625	0.616–0.634
**Progression to sepsis**	**SIC**	**0.672**	0.663–0.681
	ISTH	0.664	0.655–0.673
	EASIX	0.634	0.625–0.643
	log2-EASIX	0.634	0.625–0.643
	JAAM	0.612	0.603–0.622
**Thrombotic events**	**ISTH**	**0.614**	0.604–0.623
	JAAM	0.555	0.545–0.564
	EASIX	0.539	0.529–0.548
	log2-EASIX	0.539	0.529–0.548
	SIC	0.509	0.500–0.519
**Hemorrhagic events**	**ISTH**	**0.545**	0.535–0.554
	JAAM	0.529	0.520–0.539
	SIC	0.529	0.519–0.538
	EASIX	0.502	0.492–0.511
	log2-EASIX	0.502	0.492–0.511
**Combined coagulopathy**	**ISTH**	**0.605**	0.596–0.614
	JAAM	0.549	0.540–0.559
	EASIX	0.527	0.517–0.536
	log2-EASIX	0.527	0.517–0.536
	SIC	0.503	0.494–0.513

Receiver operating characteristic (ROC) analysis was performed in the non-overt DIC population. Best-performing scores for each outcome are shown in bold. Pairwise comparisons between ROC curves were performed using the DeLong method. Abbreviations: SIC, Sepsis-Induced Coagulopathy; JAAM, Japanese Association for Acute Medicine; ISTH, International Society on Thrombosis and Haemostasis; EASIX, Endothelial Activation and Stress Index; base-2 logarithmic transformation of the Endothelial Activation and Stress Index.

## Data Availability

The data presented in this study are available upon reasonable requests made to the corresponding author.

## Appendix A

**Table A1:** OVERT-DIC yes vs. no.

	Population	Non-OVERT DICDIC	OVERT DIC	*p*-Value
	*n* = 11,672	*n* = 11,274	*n* = 398	
**Demographics**				
Age	71 [58–81]	71 [58–81]	71 [58–80]	0.951
Charlson	4 [2–6]	4 [2–6]	5 [3–7]	**<0.001**
**Clinical parameters at presentation**				
FC	88 [77–103]	88 [77–103]	98 [81–111]	**<0.001**
GCS	15 [15–15]	15 [15–15]	15 [15–15]	0.275
SBP	132 [116–150]	133 [116–150]	120 [103–139]	**<0.001**
DBP	80 [70–90]	80 [70–90]	75 [60–84]	**<0.001**
MAP	96.67 [86.33–108.67]	96.67 [86.33–108.67]	90.00 [76.00–100.00]	**<0.001**
BT	36.4 [36–37]	36.3 [36–37]	36.5 [36.0–37.3]	**0.009**
SpO_2_	96 [92–98]	96 [92–98]	95 [91–98]	
PAO_2_	86.31 [65.73–100]	86.31 [65.73–100]	79.16 [62.67–100.00]	
FiO_2_	0.21 [0.21–0.21]	0.21 [0.21–0.21]	0.21 [0.21–0.21]	
P/F	411.01 [312.98–476.19]	411.01 [312.98–476.19]	376.94 [298.41–476.19]	0.117
**Clinical Scores**				
SIC	2.00 [0.00–2.00]	2.00 [0.00–2.00]	4.00 [4.00–5.00]	**<0.001**
JAAM2	0.00 [0.00–1.00]	0.00 [0.00–1.00]	3.00 [2.00–4.00]	**<0.001**
EASIX	1.25 [0.72–2.46]	1.20 [0.71–2.28]	11.74 [4.72–41.90]	**<0.001**
LOG2 EASIX	0.32 [−0.47–1.30]	0.26 [−0.50–1.19]	3.55 [2.24–5.39]	**<0.001**
ISTH	1.00 [0.00–3.00]	1.00 [0.00–3.00]	5.00 [5.00–5.00]	**<0.001**
**Laboratory Exams**				
HB	12.90 [11.10–14.40]	12.90 [11.10–14.40]	10.50 [8.50–13.20]	**<0.001**
RDW	14.60 [13.60–16.20]	14.60 [13.60–16.10]	16.70 [15.20–19.00]	**<0.001**
WBC	9.29 [6.84–12.80]	9.29 [6.84–12.73]	10.21 [5.43–16.25]	0.154
N/L	4.93 [2.76–9.43]	4.89 [2.75–9.34]	7.28 [3.10–12.27]	**<0.001**
PLT	227 [170–299]	230 [175–302]	57 [24–148]	
PT	11.50 [10.90–12.50]	11.50 [10.90–12.40]	17.00 [12.70–20.65]	**<0.001**
aPTT	31.00 [27.20–35.70]	30.90 [27.20–35.40]	37.40 [29.90–44.60]	**<0.001**
INR	1.07 [1.01–1.17]	1.07 [1.01–1.15]	1.60 [1.18–1.97]	**<0.001**
Fibrinogen	429 [332–586]	430 [333–585]	396 [273–614]	**0.002**
DDimer	1341 [579–3753]	1251 [561–3346]	8699 [4810–17,963]	**<0.001**
LDH	275 [209–404]	271 [208–394]	451 [302–993]	**<0.001**
Urea	19 [14–28]	19 [14–28]	31 [19–48]	**<0.001**
Creatinine	0.93 [0.73–1.27]	0.92 [0.72–1.25]	1.26 [0.86–1.97]	
Sodium	138 [135–140]	138 [135–140]	136 [132–140]	
Potassium	4.1 [3.7–4.6]	4.1 [3.7–4.6]	4.2 [3.8–4.8]	
Glucose	118 [99–150]	118 [99–150]	121 [102–146]	0.458
GPT	20 [13–33]	19 [13–33]	30 [17–68]	**<0.001**
GOT	25 [17–41]	24 [17–40]	46 [22–118]	**<0.001**
Alkaline phosphatase	85 [64–122]	83.00 [63.00–119.00]	104.00 [73.75–206.25]	**<0.001**
Bilirubin Tot	0.7 [0.5–1.0]	0.70 [0.50–1.00]	1.20 [0.76–2.43]	**<0.001**
CRP	34.00 [7.30–116.00]	32.80 [7.00–113.68]	78.05 [22.63–186.43]	
PCT	0.11 [0.05–0.43]	0.10 [0.05–0.39]	0.53 [0.14–5.40]	
NTproBNP	417.00 [0.00–2735.00]	410.00 [0.00–2644.00]	937.00 [0.00–6663.50]	
TPNUltra	0.00 [0.00–0.01]	0.00 [0.00–0.01]	0.00 [0.00–0.01]	
**Clinical Progression**				
LOS days	9.67 [6.00–16.03]	9.63 [6.00–16.00]	10.60 [5.37–21.56]	0.058

Abbreviations: DIC, disseminated intravascular coagulation; FC, heart rate; GCS, Glasgow Coma Scale; SBP, systolic blood pressure; DBP, diastolic blood pressure; MAP, mean arterial pressure; BT, body temperature; SpO_2_, peripheral oxygen saturation; PaO_2_, arterial oxygen partial pressure; FiO_2_, fractional inspired oxygen; P/F, PaO_2_/FiO_2_ ratio; SIC, Sepsis-Induced Coagulopathy; JAAM2, Japanese Association for Acute Medicine2; EASIX, Endothelial Activation and Stress Index; log2-EASIX, base-2 logarithmic transformation of the Endothelial Activation and Stress Index; ISTH, International Society on Thrombosis and Haemostasis; HB, hemoglobin; RDW, red cell distribution width; WBC, white blood cell count; N/L, neutrophil-to-lymphocyte ratio; PLT, platelet count; PT, prothrombin time; aPTT, activated partial thromboplastin time; INR, international normalized ratio; LDH, lactate dehydrogenase; GPT, glutamate pyruvate transaminase; GOT, glutamate oxaloacetate transaminase; CRP, C-reactive protein; PCT, procalcitonin; NT-proBNP, N-terminal pro-B-type natriuretic peptide; TPN Ultra, high-sensitivity troponin; LOS, length of stay. Data are expressed as median [IQR] unless otherwise specified.

**Table A2:** Non-OVERT DIC patients. Endpoint: death.

	Population [Non-OVERT DIC]	Death No	Death Yes	*p*–Value
	*n* = 11,274	*n* = 10,000	*n* = 1274	
**Demographics**				
Age	71 [58–81]	70 [57–80]	78 [69–85]	**<0.001**
Charlson	4 [2–6]	4 [2–6]	6 [4–7]	**<0.001**
**Clinical parameters at presentation**				
FC	88 [77–103]	88 [77–102]	91 [80–110]	**<0.001**
GCS	15 [15–15]	15 [15–15]	15 [15–15]	**<0.001**
SBP	133 [116–150]	134 [119–150]	125 [104–142]	**<0.001**
DBP	80 [70–90]	80 [70–90]	74 [61–86]	**<0.001**
MAP	96.67 [86.33–108.67]	97.67 [87–109.67]	92 [78–103.33]	**<0.001**
BT	36.3 [36–37]	36.3 [36.0–37.0]	36.4 [36.0–37.1]	0.135
SpO_2_	96 [92–98]	96 [92–98]	92 [87–96]	**<0.001**
PAO_2_	86.31 [65.73–100]	86.31 [65.73–112.43]	65.73 [53.78–86.31]	**<0.001**
FiO_2_	0.21 [0.21–0.21]	0.21 [0.21–0.21]	0.21 [0.21–0.21]	**<0.001**
P/F	411.01 [312.98–476.19]	411.01 [312.98–535.38]	312.98 [248.11–411.01]	**<0.001**
**Clinical Scores**				
SIC	1.00 [0.00–2.00]	1.00 [0.00–2.00]	2.00 [2.00–3.00]	**<0.001**
JAAM2	0.00 [0.00–1.00]	0.00 [0.00–1.00]	1.00 [0.00–1.00]	**<0.001**
EASIX	1.20 [0.71–2.28]	1.15 [0.69–2.06]	2.17 [1.01–4.58]	**<0.001**
LOG2 EASIX	0.26 [−0.50–1.19]	0.20 [−0.54–1.06]	1.12 [0.02–2.20]	**<0.001**
ISTH	1.00 [0.00–3.00]	0.00 [0.00–2.00]	2.00 [0.00–3.00]	**<0.001**
**Laboratory Exams**				
HB	12.90 [11.10–14.40]	13.00 [11.30–14.50]	11.80 [10.30–13.60]	**<0.001**
RDW	14.60 [13.60–16.10]	14.50 [13.50–15.90]	15.60 [14.30–17.40]	**<0.001**
WBC	9.29 [6.84–12.73]	9.10 [6.79–12.34]	11.12 [7.47–15.74]	**<0.001**
N/L	4.89 [2.75–9.34]	4.59 [2.63–8.56]	8.84 [4.84–15.77]	**<0.001**
PLT	230 [175–302]	231 [177–300]	221 [154–326]	**<0.001**
PT	11.50 [10.90–12.40]	11.40 [10.90–12.30]	12.20 [11.40–13.50]	**0.035**
aPTT	30.90 [27.20–35.40]	30.80 [27.10–35.10]	32.30 [27.60–38.10]	**<0.001**
INR	1.07 [1.01–1.15]	1.06 [1.01–1.14]	1.13 [1.05–1.26]	
Sodium	138 [135–140]	138.00 [135.00–141.00]	137.00 [133.00–140.00]	**<0.001**
Potassium	4.1 [3.7–4.6]	4.1 [3.7–4.5]	4.3 [3.8–5.0]	**<0.001**
Glucose	118 [99–150]	118 [99–150]	118 [98–146]	0.226
GPT	19 [13–33]	19 [13–32]	21 [13–38]	**0.001**
GOT	24 [17–40]	24 [17–38]	33.00 [20–61]	**<0.001**
Alkaline phosphatase	83.00 [63.00–119.00]	82.00 [63.00–116.00]	93.00 [69.00–170.00]	**<0.001**
Bilirubin Tot	0.70 [0.50–1.00]	0.70 [0.50–1.00]	0.70 [0.50–1.10]	0.173
CRP	32.80 [7.00–113.68]	28.40 [5.90–104.10]	85.30 [27.90–157.08]	**<0.001**
PCT	0.10 [0.05–0.39]	0.09 [0.05–0.31]	0.23 [0.08–0.90]	**<0.001**
**Clinical Progression**				
LOS days	9.63 [6.00–16.00]	9.75 [6.17–15.71]	9.00 [3.58–18.93]	**<0.001**

Abbreviations: BT, body temperature; CRP, C-reactive protein; DBP, diastolic blood pressure; EASIX, Endothelial Activation and Stress Index; FC, heart rate; FiO_2_, fraction of inspired oxygen; GCS, Glasgow Coma Scale; GPT, glutamate pyruvate transaminase (ALT); GOT, glutamate oxaloacetate transaminase (AST); HB, hemoglobin; INR, international normalized ratio; ISTH, International Society on Thrombosis and Haemostasis DIC score; JAAM2, Japanese Association for Acute Medicine DIC score; LOS, length of stay; log2-EASIX, base-2 logarithmic transformation of EASIX; MAP, mean arterial pressure; N/L, neutrophil-to-lymphocyte ratio; PaO_2_, partial pressure of arterial oxygen; P/F, PaO_2_/FiO_2_ ratio; PCT, procalcitonin; PLT, platelet count; PT, prothrombin time; RDW, red cell distribution width; SBP, systolic blood pressure; SIC, Sepsis-Induced Coagulopathy score; SpO_2_, peripheral oxygen saturation; WBC, white blood cell count. Data are expressed as median [IQR] unless otherwise specified.

**Table A3:** Non-OVERT DIC patients. Endpoint: coagulopathy.

	Population [Non-OVERT DIC]	Coagulopathy No	Coagulopathy Yes	*p*–Value
	*n* = 11,274	*n* = 7944	*n* = 3330	
**Demographics**				
Age	71 [58–81]	70 [57–80]	73 [61–82]	**<0.001**
Charlson	4 [2–6]	4 [2–6]	4 [3–6]	**<0.001**
**Clinical parameters at presentation**				
FC	88 [77–103]	89 [78–103]	87 [75–101]	**<0.001**
GCS	15 [15–15]	15 [15–15]	15 [15–15]	0.670
SBP	133 [116–150]	132 [117–150]	133.00 [116–150]	0.497
DBP	80 [70–90]	80 [70–90]	80 [70–90]	0.203
MAP	96.67 [86.33–108.67]	96.67 [86.67–108.67]	96.67 [86.67–109.00]	0.646
BT	36.3 [36–37]	36.40 [36–37]	36.20 [36–37]	**<0.001**
SpO_2_	96 [92–98]	96 [91–98]	96 [93–98]	**<0.001**
PAO_2_	86.31 [65.73–100]	86.31 [62.67–100.00]	86.31 [69.33–112.43]	**<0.001**
FiO_2_	0.21 [0.21–0.21]	0.21 [0.21–0.21]	0.21 [0.21–0.21]	0.569
P/F	411.01 [312.98–476.19]	411.01 [298.41–476.19]	411.01 [312.98–535.38]	**<0.001**
**Clinical Scores**				
SIC	1.00 [0.00–2.00]	2.00 [0.00–2.00]	2.00 [0.00–2.00]	0.427
JAAM2	0.00 [0.00–1.00]	0.00 [0.00–1.00]	0.00 [0.00–1.00]	**<0.001**
EASIX	1.20 [0.71–2.28]	1.17 [0.69–2.21]	1.28 [0.75–2.43]	**<0.001**
LOG2 EASIX	0.26 [−0.50–1.19]	0.23 [−0.53–1.15]	0.35 [−0.41–1.28]	**<0.001**
ISTH	1.00 [0.00–3.00]	0.00 [0.00–2.00]	2.00 [0.00–3.00]	**<0.001**
**Laboratory Exams**				
HB	12.90 [11.10–14.40]	13.00 [11.30–14.40]	12.70 [11.00–14.20]	**<0.001**
RDW	14.60 [13.60–16.10]	14.50 [13.50–16.00]	14.80 [13.70–16.40]	**<0.001**
WBC	9.29 [6.84–12.73]	9.25 [6.79–12.77]	9.29 [6.97–12.65]	0.545
N/L	4.89 [2.75–9.34]	4.93 [2.75–9.42]	4.79 [2.78–9.07]	0.452
PLT	230 [175–302]	231 [176–302]	226 [172–301]	**0.025**
PT	11.50 [10.90–12.40]	11.50 [10.90–12.40]	11.50 [11.00–12.40]	0.092
aPTT	30.90 [27.20–35.40]	31.30 [27.50–35.70]	29.90 [26.30–34.60]	**<0.001**
INR	1.07 [1.01–1.15]	1.07 [1.01–1.15]	1.07 [1.01–1.16]	0.430
Glucose	118 [99–150]	119 [99–150]	117 [99–150]	0.645
GPT	19 [13–33]	20 [13–33]	19 [13–31]	**0.013**
GOT	24 [17–40]	25 [17–42]	23 [16–36]	**<0.001**
Alkaline phosphatase	83.00 [63.00–119.00]	83.00 [63.00–118.00]	84.00 [65.00–120.00]	0.567
Bilirubin Tot	0.70 [0.50–1.00]	0.70 [0.50–1.00]	0.70 [0.50–1.00]	0.755
CRP	32.80 [7.00–113.68]	35.30 [7.20–118.18]	28.10 [6.50–102.38]	**<0.001**
PCT	0.10 [0.05–0.39]	0.10 [0.05–0.41]	0.10 [0.05–0.34]	0.105
NTproBNP	410.00 [0.00–2644.00]	388.00 [0.00–2551.75]	468.00 [0.00–2835.25]	
TPNUltra	0.00 [0.00–0.01]	0.00 [0.00–0.01]	0.00 [0.00–0.01]	
**Clinical Progression**				
LOS days	9.63 [6.00–16.00]	9.55 [6.00–15.64]	10.00 [6.11–16.62]	**<0.001**

Abbreviations: aPTT, activated partial thromboplastin time; BT, body temperature; CRP, C-reactive protein; DBP, diastolic blood pressure; EASIX, Endothelial Activation and Stress Index; FC, heart rate; FiO_2_, fraction of inspired oxygen; GCS, Glasgow Coma Scale; GPT, glutamate pyruvate transaminase (ALT); GOT, glutamate oxaloacetate transaminase (AST); HB, hemoglobin; INR, international normalized ratio; ISTH, International Society on Thrombosis and Haemostasis DIC score; JAAM2, Japanese Association for Acute Medicine DIC score; LOS, length of stay; log2-EASIX, base-2 logarithmic transformation of EASIX; MAP, mean arterial pressure; N/L, neutrophil-to-lymphocyte ratio; NT-proBNP, N-terminal pro-B-type natriuretic peptide; PaO_2_, partial pressure of arterial oxygen; P/F, PaO_2_/FiO_2_ ratio; PCT, procalcitonin; PLT, platelet count; PT, prothrombin time; RDW, red cell distribution width; SBP, systolic blood pressure; SIC, Sepsis-Induced Coagulopathy score; SpO_2_, peripheral oxygen saturation; TPN Ultra, high-sensitivity troponin T; WBC, white blood cell count. Data are expressed as median [IQR] unless otherwise specified.

**Table A4:** Non-OVERT-DIC, only septic patients. Endpoint: coagulopathy.

	All Septic Patients	Coagulopathy NO	Coagulopathy YES	*p*–Value
	*n* = 663	*n* = 494	*n* = 169	
**Demographics**				
Age	74.00 [64.00–83.00]	74.00 [64.00–82.00]	77.00 [64.00–83.00]	0.376
Charlson	5.00 [3.00–6.00]	5.00 [3.00–6.00]	5.00 [4.00–6.50]	0.119
**Clinical parameters at presentation**				
FC	95.00 [80.00–110.00]	95.00 [81.25–112.00]	90.00 [79.00–105.00]	0.093
GCS	15.00 [15.00–15.00]	15.00 [15.00–15.00]	15.00 [15.00–15.00	0.923
SBP	121.00 [101.00–140.00]	120.00 [100.00–140.00]	125.50 [105.00–144.25]	0.205
DBP	70.00 [60.00–82.00]	70.00 [60.00–81.00]	72.00 [60.00–85.00]	0.278
MAP	88.83 [76.00–101.67]	88.67 [75.75–100.00]	90.00 [76.50–105.80]	0.177
BT	36.70 [36.00–37.80]	36.80 [36.00–37.80]	36.50 [36.50–37.60]	0.214
SpO_2_	95.00 [90.00–97.00]	94.00 [90.00–97.00]	95.00 [92.00–97.00]	**0.034**
PAO_2_	79.16 [60.02–96.39]	73.70 [60.02–96.39]	79.16 [65.73–96.39]	**0.046**
FiO_2_	0.21 [0.21–0.21]	0.21 [0.21–0.21]	0.21 [0.21–0.21]	0.581
P/F	376.94 [285.92–458.98]	350.95 [285.82–458.98]	376.94 [298.41–458.98]	**0.049**
**Clinical Scores**				
SIC	2.00 [2.00–3.00]	2.00 [2.00–3.00]	2.00 [1.00–3.00]	**0.018**
JAAM2	0.00 [0.00–1.00]	0.50 [0.00–1.00]	0.00 [0.00–1.00]	0.737
EASIX	1.92 [0.98–4.44]	1.87 [1.01–4.45]	1.97 [0.91–4.57]	0.838
LOG2 EASIX	0.94 [−0.03–2.15]	0.91 [0.00–2.15]	1.00 [−0.13–2.19]	0.838
ISTH	2.00 [1.00–3.00]	2.00 [1.00–3.00]	2.00 [1.00–3.00]	0.993
**Laboratory Exams**				
HB	11.60 [10.00–13.20]	11.60 [10.00–13.20]	11.60 [9.65–13.06]	0.733
RDW	15.40 [14.20–17.10]	15.40 [14.18–17.00]	15.60 [14.45–17.40]	0.128
WBC	11.43 [7.58–16.19]	11.45 [7.63–16.42]	11.40 [7.36–15.26]	0.483
N/L	10.66 [5.72–20.07]	10.96 [6.22–20.58]	9.49 [4.50–18.21]	0.054
PLT	206.00 [142.00–291.00]	205.50 [140.75–287.00]	206.00 [144.50–316.25]	0.657
PT	12.00 [11.40–13.20]	12.00 [11.40–13.40]	11.80 [11.30–12.80]	0.073
aPTT	33.30 [29.00–38.60]	33.70 [29.55–38.95]	31.60 [27.65–37.35]	**0.005**
INR	1.13 [1.06–1.24]	1.13 [1.06–1.25]	1.11 [1.06–1.2]	0.064
Fibrinogen	550.00 [396.00–731.00]	561.00 [408.50–747.00]	507.50 [362.75–694.00]	**0.039**
DDimer	2766.00 [1258.00–5649.00]	2696.00 [1235.00–5374.00]	3053.00 [1385.25–6608.00	0.236
LDH	298.00 [216.00–431.00]	296.00 [216.00–430.00]	301.00 [214.75–443.00]	0.815
Urea	27.00 [18.00–41.00]	27.00 [18.00–40.00]	28.00 [18.00–42.00]	0.879
Creatinine	1.18 [0.80–1.88]	1.19 [0.81–1.87]	1.15 [0.78–1.93]	0.665
Sodium	137.00 [133.00–140.00]	136.00 [133.00–140.00]	137.00 [132.75–141.00]	0.089
Potassium	4.10 [3.60–4.80]	4.10 [3.60–4.85]	4.20 [3.70–4.80]	0.322
**Clinical Progression**				
LOS days	15.68 [9.57–28.10]	15.00 [9.24–26.50]	17.59 [10.46–30.50]	0.055

Abbreviations: aPTT, activated partial thromboplastin time; BT, body temperature; DBP, diastolic blood pressure; DDimer, D-dimer; EASIX, Endothelial Activation and Stress Index; FC, heart rate; FiO_2_, fraction of inspired oxygen; GCS, Glasgow Coma Scale; HB, hemoglobin; INR, international normalized ratio; ISTH, International Society on Thrombosis and Haemostasis DIC score; JAAM2, Japanese Association for Acute Medicine DIC score; LDH, lactate dehydrogenase; log2-EASIX, base-2 logarithmic transformation of EASIX; LOS, length of stay; MAP, mean arterial pressure; N/L, neutrophil-to-lymphocyte ratio; PaO_2_, partial pressure of arterial oxygen; P/F, PaO_2_/FiO_2_ ratio; PLT, platelet count; PT, prothrombin time; RDW, red cell distribution width; SBP, systolic blood pressure; SIC, Sepsis-Induced Coagulopathy score; SpO_2_, peripheral oxygen saturation; WBC, white blood cell count. Data are expressed as median [IQR] unless otherwise specified.

**Table A5:** Non-OVERT-DIC, only septic patients. Endpoint: death.

	All Septic Patients	Death NO	Death YES	*p*–Value
	*n* = 663	*n* = 499	*n* = 166	
**Demographics**				
Age	74.00 [64.00–83.00]	73.00 [63.00–82.00]	78.00 [70.00–86.00]	**<0.001**
Charlson	5.00 [3.00–6.00]	5.00 [3.00–6.00]	5.00 [4.00–7.00]	**0.008**
**Clinical parameters at presentation**				
FC	95.00 [80.00–110.00]	95.00 [81.00–110.00]	90.00 [78.50–111.75]	0.493
GCS	15.00 [15.00–15.00]	15.00 [15.00–15.00]	15.00 [15.00–15.00]	0.078
SBP	121.00 [101.00–140.00]	124.00 [104.00–140.00]	120.00 [100.00–136.00]	**0.019**
DBP	70.00 [60.00–82.00]	71.00 [60.00–83.00]	70.00 [60.00–80.00]	0.078
MAP	88.83 [76.00–101.67]	90.00 [76.67–103.00]	85.33 [73.3–99.00]	**0.019**
BT	36.70 [36.00–37.80]	36.80 [36.00–37.90]	36.70 [36.00–37.50]	0.182
**Clinical Scores**				
SIC	2.00 [2.00–3.00]	2.00 [1.00–3.00]	2.00 [2.00–3.00]	**0.008**
JAAM2	0.00 [0.00–1.00]	0.00 [0.00–1.00]	1.00 [0.00–1.00]	0.089
EASIX	1.92 [0.98–4.44]	1.78 [0.97–4.04]	2.86 [1.09–6.21]	**0.006**
LOG2 EASIX	0.94 [−0.03–2.15]	0.83 [−0.05–2.06]	1.52 [0.12–2.64]	**0.006**
ISTH	2.00 [1.00–3.00]	2.00 [0.00–3.00]	3.00 [2.00–3.00]	0.064
**Laboratory Exams**				
N/L	10.66 [5.72–20.07]	10.42 [5.64–19.68]	11.70 [6.22–20.78]	0.267
PLT	206.00 [142.00–291.00]	201.00 [140.00–283.00]	223.00 [159.50–332.00]	**0.038**
PT	12.00 [11.40–13.20]	11.90 [11.30–13.40]	12.20 [11.40–14.30]	**0.007**
aPTT	33.30 [29.00–38.60]	33.30 [29.00–38.40]	33.20 [29.08–39.75]	0.459
INR	1.13 [1.06–1.24]	1.12 [1.06–1.23]	1.15 [1.07–1.34]	**0.020**
Fibrinogen	550.00 [396.00–731.00]	560.00 [408.00–745.00]	509.00 [371.00–698.00]	**0.019**
DDimer	2766.00 [1258.00–5649.00]	2458.00 [1214.00–5171.50]	3146.50 [1395.00–7200.00	**0.017**
LDH	298.00 [216.00–431.00]	279.00 [207.25–402.25]	342.00 [259.00–516.00]	**<0.001**
Urea	27.00 [18.00–41.00]	25.00 [17.00–38.00]	32.00 [21.00–49.25]	**<0.001**
NTproBNP	91.00 [0.00–5238.00]	690.00 [0.00–4197.00]	2424.00 [166.7–8503.25]	**<0.001**
TPNUltra	0.00 [0.00–0.00]	0.00 [0.00–0.01]	0.00 [0.00–0.01]	0.273
**Clinical Progression**				
LOS days	15.68 [9.57–28.10]	15.9 [10.26–28.16]	14.93 [5.39–26.35]	**0.002**

Abbreviations: aPTT, activated partial thromboplastin time; BT, body temperature; DBP, diastolic blood pressure; DDimer, D-dimer; EASIX, Endothelial Activation and Stress Index; FC, heart rate; GCS, Glasgow Coma Scale; INR, international normalized ratio; ISTH, International Society on Thrombosis and Haemostasis DIC score; JAAM2, Japanese Association for Acute Medicine DIC score; LDH, lactate dehydrogenase; log2-EASIX, base-2 logarithmic transformation of EASIX; LOS, length of stay; MAP, mean arterial pressure; N/L, neutrophil-to-lymphocyte ratio; NT-proBNP, N-terminal pro-B-type natriuretic peptide; PLT, platelet count; PT, prothrombin time; SBP, systolic blood pressure; SIC, Sepsis-Induced Coagulopathy score; TPNUltra, high-sensitivity troponin T. Data are expressed as median [IQR] unless otherwise specified.
